# Effects of Ocean Acidification on Juvenile Red King Crab (*Paralithodes camtschaticus*) and Tanner Crab (*Chionoecetes bairdi*) Growth, Condition, Calcification, and Survival

**DOI:** 10.1371/journal.pone.0060959

**Published:** 2013-04-04

**Authors:** William Christopher Long, Katherine M. Swiney, Caitlin Harris, Heather N. Page, Robert J. Foy

**Affiliations:** Kodiak Laboratory, Resource Assessment and Conservation Engineering Division, Alaska Fisheries Science Center, National Marine Fisheries Service, NOAA, Kodiak, Alaska, United States of America; University of Gothenburg, Sweden

## Abstract

Ocean acidification, a decrease in the pH in marine waters associated with rising atmospheric CO_2_ levels, is a serious threat to marine ecosystems. In this paper, we determine the effects of long-term exposure to near-future levels of ocean acidification on the growth, condition, calcification, and survival of juvenile red king crabs, *Paralithodes camtschaticus,* and Tanner crabs, *Chionoecetes bairdi*. Juveniles were reared in individual containers for nearly 200 days in flowing control (pH 8.0), pH 7.8, and pH 7.5 seawater at ambient temperatures (range 4.4–11.9 °C). In both species, survival decreased with pH, with 100% mortality of red king crabs occurring after 95 days in pH 7.5 water. Though the morphology of neither species was affected by acidification, both species grew slower in acidified water. At the end of the experiment, calcium concentration was measured in each crab and the dry mass and condition index of each crab were determined. Ocean acidification did not affect the calcium content of red king crab but did decrease the condition index, while it had the opposite effect on Tanner crabs, decreasing calcium content but leaving the condition index unchanged. This suggests that red king crab may be able to maintain calcification rates, but at a high energetic cost. The decrease in survival and growth of each species is likely to have a serious negative effect on their populations in the absence of evolutionary adaptation or acclimatization over the coming decades.

## Introduction

Since the beginning of the Industrial Revolution, CO_2_ release from anthropogenic activities such as the burning of fossil fuels and the manufacturing of concrete has resulted in increasing atmospheric CO_2_ concentration [1]. This has led to a commensurate increase in the CO_2_ concentration in seawater, resulting in a shift in the carbonate chemistry and a decrease in pH known as ocean acidification [2]. The pH of oceans globally has decreased by about 0.1 units since the Industrial Revolution and is projected to decrease even further in the future [3]. The change in water chemistry has an effect on many of the species living in the oceans and is likely to cause substantial changes in marine ecosystems [1], [4].

Ocean acidification typically has either a negative or neutral effect on most marine animals [5]. Calcifying organisms, including corals, bivalves, gastropods, echinoderms, and crustaceans, are thought to be particularly vulnerable, as a lower pH increases the dissolution rates of calcium carbonate [6]–[9]. Additionally, the embryos and larvae of some species exhibit lower survival rates [10], [11], lower growth rates [12], [13], increased rates of malformation [14], [15], and even changes in behavior [16] under acidified conditions. On the other hand, many species show little to no effect of ocean acidification [17]–[20], and others show positive effects (e.g. [21], [22]). Some species, such as those adapted to live in estuaries or in the intertidal, are already adapted to live in a constantly fluctuating environment and are therefore relatively robust in the face of pH changes, whereas others that are adapted to live in a more constant environment are less able to adapt to acidified water [23]. Although many studies on the effects of future pH decreases have been done, most have a fairly short experimental duration (e.g., [24], [25], [26]). Longer term studies are needed, as the short-term effects do not necessarily predict the long-term ones [27].

In this study, we examine the effects of ocean acidification on the growth, condition, calcification, and survival of juveniles of two commercially important and phylogenetically distinct species of crab from the Bering Sea and Gulf of Alaska. Red king crab (RKC), *Paralithodes camtschaticus*, is an Anomuran crab species, and the Tanner crab, *Chionoecetes bairdi*, is a Brachyuran species. The species are marine and have similar ranges and distributions but juveniles differ in their micro-habitat use; red king crabs prefer structured habitat [28], probably because they decrease predation risk [29], [30] whereas Tanners prefer unstructured soft sediment [31]. However, given that both are benthic and their ranges overlap so substantially, there is no reason to suspect that these habitats differ in terms of pH or carbonate chemistry. Both species brood eggs for about a year before releasing larvae into the water column in the spring, typically between February and May [32]–[34]. The larvae spend several months as plankton before settling to benthic habitat, where they grow to maturity in about 5–7 years [35], [36]. Tanner crabs molt about 6 times in their first year with molting rates decreasing with crab size [37]. Both male and female Tanner crabs undergo terminal molts [38], [39] which for males may take place after18 molts and 12 years [35]. The maximum age of Tanner crabs is unknown but estimates range from 14–20 y [40]. Red king crabs are estimated to molt 8 times in their first year post-settlement [37] with molting rates decreasing to once per year for mature females [41] and even lower frequency for large males [42]. As there is no method to age red king crab, their maximum age is unknown, but is likely at least 20 years [42]. In crabs, calcification occurs internally [43]. The time it takes to harden completely varies with species and size but it can take a substantial amount of time; mature red king crab females, for example take about 74 days to harden after a molt [44].

As northern latitude waters are expected to experience a greater rate and degree of acidification than tropical waters, understanding the species-specific responses to ocean acidification in this ecosystem is important [45]. Red king crab embryos and larvae are sensitive to even small changes in pH [46] and initial research suggests that Tanner crab embryos and larvae are tolerant to changes in pH (WCL, KWS, RJF unpublished data). In addition we conducted this study over a long time period, in order to differentiate between the short and long-term effects of exposure to acidified water [27].

## Materials and Methods

### Ethics Statement

Ethical approval for this research was not required by any federal, state, or international law because the animals used were invertebrates and therefore not covered. The transportation and field collection of the animals was authorized by the Alaska Department of Fish and Game (Fish Transport Permit 10A-1045 and Fish Resource permits numbers CF-10-074 and CF-10-075).

For this experiment, we used filtered seawater pumped into the laboratory from Trident Basin (Kodiak, AK). The experimental setup used flow-through water at ambient temperature and salinity. As we did not control temperature, the conditions the crabs were exposed to mimicked the natural fluctuations to which crabs would naturally be exposed. We used seawater acidified with CO_2_ to pHs based on projected future levels of atmospheric CO_2_ and the predicted change in seawater pH associated with it: 1) ambient pH (∼8.0), 2) 7.8 pH c. 2100, and 3) 7.5 pH c. 2200 [3]. The ambient water used in this experiment was obtained at a depth at which both species occur and so is representative of what these species experience. To obtain the desired treatment levels CO_2_ was bubbled in ambient local seawater to a pH of 5.5. This water was mixed with seawater to the treatment pHs using a peristaltic pump whose speed was controlled by a pH probe in a head tank similar to the design by McGraw et al. [47]. Water from the head tank was supplied to the experimental containers. When the measured pH in the experimental containers deviated from the nominal pH levels by more than 0.02 units the settings on the pH probe were adjusted accordingly.

Red king crabs were supplied by the Alutiiq Pride Shellfish Hatchery. Ovigerous red king crabs were captured in Bristol Bay, Alaska, in commercial pots during the winter of 2009. Larvae were reared to the first crab stage before being transported to the Kodiak lab in insulated shipping containers. Juvenile Tanner crabs were caught in a modified benthic sled with a 1 m mouth opening in local Kodiak waters. Tanner crabs were newly settled, probably at the first or second crab stage. Throughout the experiment, the crabs were fed to excess on a gel diet of “Gelly Belly” (Florida Aqua Farms, Inc., Dade City, Florida, USA; Use of trade names does not imply endorsement by the National Marine Fisheries Service, NOAA) enhanced with Cyclop-eeze powder and pollock bone powder (United States Department of Agriculture, Agricultural Research Service, Kodiak, Alaska, USA). Crabs were fed three times a week and old food was removed just prior to feeding.

The experiment was performed in three tanks (120 (L) x 60 (W) x 60 (H) cm), each of which was randomly assigned a treatment. Ninety crabs per species were randomly assigned to each of three treatments (30 crabs per species per treatment). Each crab was placed in an individual holding cell made of a piece of PVC pipe (diameter 5.1 cm) with mesh glued on the bottom. These cells are large enough that they should not limit the growth of juvenile red king crab of this size [48]. Flow-through water from the head tanks was provided to each cell. The Tanner crab experiment was started on June 4, 2010, and the red king crab experiment on June 10, 2010. Daily, five randomly selected cells per treatment per species were monitored for pH and temperature. pH was measured using a Ross Combination glass bulb pH electrode (Thermo Electron Corporation, Beverly, MA) calibrated with Tris buffer on the pH_F_ scale according to Millero [49]. Weekly water samples were taken from the head tanks, poisoned with mercuric chloride, and sent to an analytical laboratory for dissolved inorganic carbon (DIC) and alkalinity analysis. DIC was determined using a CM5014 Coulometer with a CM5130 Acidification Module (UIC Inc., Joliet, IL) using Certified Reference Material from the Dickson Laboratory (Scripps Institute, San Diego, CA) [50]. Alkalinity was measured via open cell titration according to the procedure in Dickenson et al. [50]. The pH, temperature, DIC and alkalinity were analyzed with a one-way analysis (ANOVA) of variance (SYSTAT 12.00.08, Chicago, Illinois USA). In this and in all ANOVA tests we verified the assumption of homogeneity of variance with Levene's test. Where there was a significant effect, Fisher's least-significant difference test was used to examine differences within the factors. The measured temperature, DIC, and pH were used to calculate pCO_2_, HCO_3_^−^, CO_3_^−2^, Ω_Aragonite_, and Ω_Calcite_ using the seacarb package in R [51] (R 2.14.0, Vienna, Austria).

**Figure 1:**
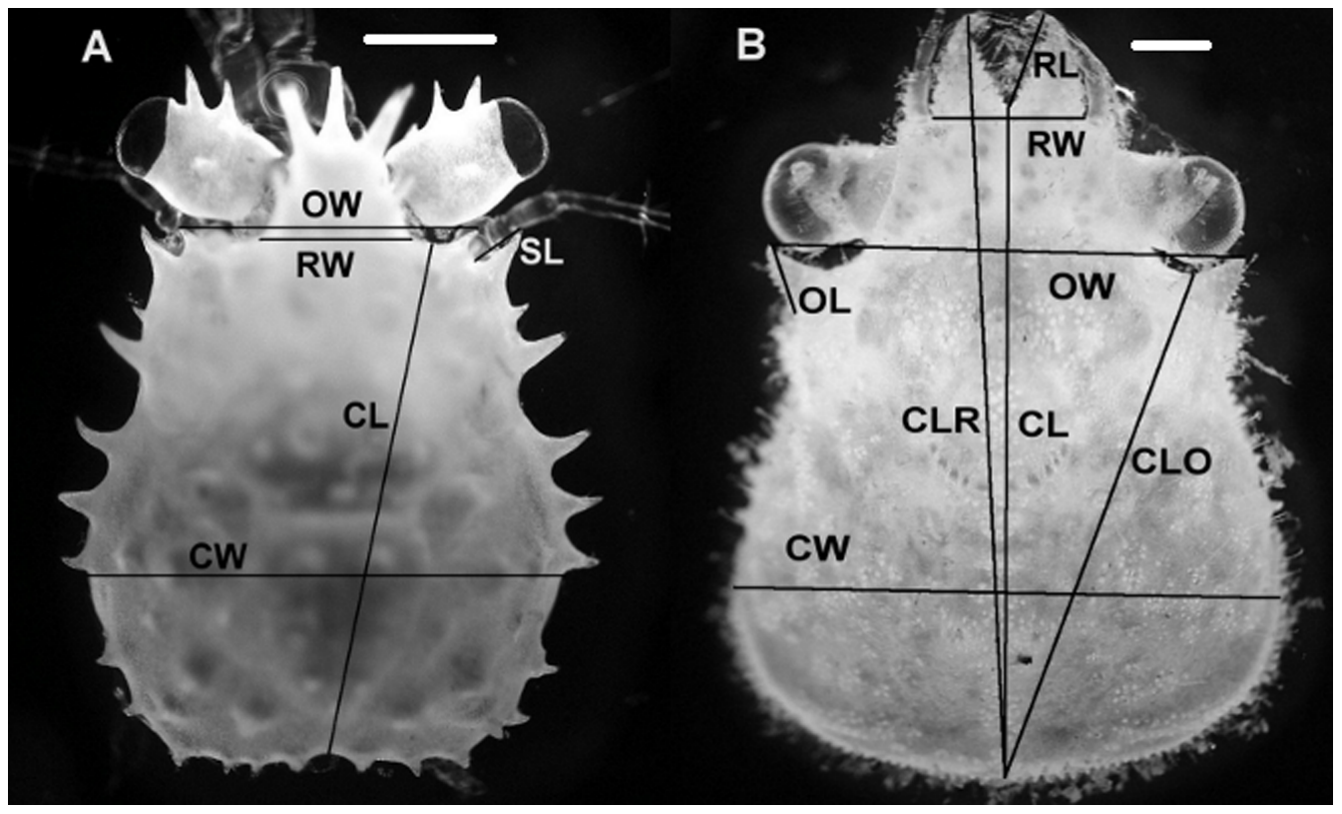
Measurement taken for morphometric analysis. A) red king crabs, and B) Tanner crabs. Measurement on red king crabs included carapace width (CW), carapace length (CL), rostrum base width (RW), orbital spine width (OW), and the first spine length (SL). Measurements on Tanner crab included carapace width (CW), carapace length (CL), carapace length to the rostrum (CLR), carapace length to the eye orbit (CLO), rostrum base width (RW), rostrum length (RL), orbital spine width (OW), and orbital spine length (OL). The scale bars are 0.5 mm.

Crabs were checked daily for molting or death. Dead crabs and exuvia were removed from the tanks for morphometric analysis. The carapace from each exuvia and dead crab was carefully removed and photographed under a stereomicroscope. Partway through the experiment, we noted that it had become difficult to remove the carapace off dead crabs, particularly in the low pH treatments, so we started photographing dead crabs before attempting to remove the carapace. If successful, we photographed the carapace as well and used that for image analysis; otherwise, we used the image of the dead crab. Image analysis was performed using Image-Pro Plus v. 6.00.260 imaging software (Media Cybernetics, Inc., Bethesda, Maryland, USA) calibrated with a micrometer photograph. On red king crab, we measured carapace width, carapace length, rostrum base width, orbital spine width, and the first spine length (Fig. 1). On Tanner crab, we measured carapace width (CW), carapace length (CL), carapace length to the rostrum, carapace length to the eye orbit, rostrum base width, rostrum length, orbital spine width, and orbital spine length (Fig. 1). The wet mass of each crab, after it was carefully blotted dry, was measured at the beginning of the experiment and 7 days after each molt.

The experiments were ended on December 20, 2010, when temperatures had dropped low enough that the crabs were no longer molting frequently. Total experimental duration was 199 days for Tanner crabs and 192 days for red king crab. At the end of the experiment, all crabs were sacrificed by freezing. The crabs were imaged for morphometric analysis as above. Each crab was dried to a constant mass at 60 °C to obtain the dry mass. Calcium and magnesium content in each crab was determined at an analytical laboratory using a Dionex Ion Chromatography system.

Mortality was modeled for each species as a binomial probability distribution. The mortality rate was assumed to be constant such that: *p^M^* = *e^−rt^*, where *p_M_* is the probability of mortality, *r* is the mortality rate, and *t* is the time in days. We used maximum likelihood to fit the data in R 2.9.2 to a series of three models in which the mortality rate was 1) the same in all treatments, 2) lower in the control than in the two low pH treatments, and 3) different in all three treatments. The Akaike's information criterion, corrected for small sample size (AIC_c_), was calculated for each model and the best model was selected. Here, and in all places where we use the AIC_c_ to rank models, we consider models whose AIC_c_s differed by less that 2 to explain the data equally well [52].

Juvenile morphometric data were analyzed with principal component analysis (PCA) in Primer 6.1.13 (Plymouth, UK). Measurements were normalized (i.e. expressed in terms of their standard deviation from the mean) prior to analysis. Differences in the principal components (PC) were analyzed with a general linear model with Treatment fully crossed with Molt number and Crab nested within Treatment as factors. Where there was a significant effect, Fisher's least-significant difference test was used to examine differences within the factors.

Red king crab growth was analyzed as a change in CL and a change in wet mass (WM) over time. As it was impossible to know where the crabs were in the molt cycle at the beginning of the experiment, the initial size was not included in analysis; however, most crabs molted within the first week of the experiment. Only crabs that had at least three data points were used. As no crab in the pH 7.5 treatment molted more than twice before dying, they were not analyzed. Change in CL over time was modeled as a linear model [35] and was analyzed with an analysis of covariance with Treatment fully crossed with Time and Crab number nested within Treatment as factors. A significant interaction between Treatment and Time was interpreted as indicating a difference in growth rates and when that occurred the treatments were analyzed separately with Time and Crab number as factors. Change in wet mass over time was modeled as an exponential increase such that 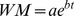, where *WM* is the wet mass, *a* and *b* are parameters, and *t* is time in degree days. Degree days are typically used when modeling crab growth to account for the effect of temperature (e.g. [36]). We fit the data to a series of models using maximum likelihood assuming a normal distribution of errors: 1) No difference between treatments, 2) *a* differed among treatments, 3) *b* differed among treatments, and 4) *a* and *b* differed among treatments. In all models, differences among crabs within a treatment were modeled by allowing *a* to vary with crab number. We calculated the AIC_c_ for each model and used that to rank the models and select the best one.

Growth in Tanner crab could not be directly analyzed as a function of time, as the RKC were, because too few crabs molted three times during the experiment. We analyzed the initial CW and WM, the CW and WM after the first and second molts, the CW and WM at the end of the experiment, the intermolt duration before the first molt, and the intermolt duration between the first and second molt (in degree days) with ANOVAs with Treatment as the factor. Where Levene's test indicated heteroscedastic data, we used a Kruskal-Wallis (KW) test instead.

The conditions of the crab at the end of the experiment were calculated as the condition index (also known as the body mass index) defined as the dry mass in grams divided by the CL^3^ (red king crab) or CW^3^ (Tanners) in millimeters (e.g. [53]). The condition index and the percent calcium and magnesium were analyzed with a one-way ANOVA with Treatment as the factor.

## Results

**Figure 2:**
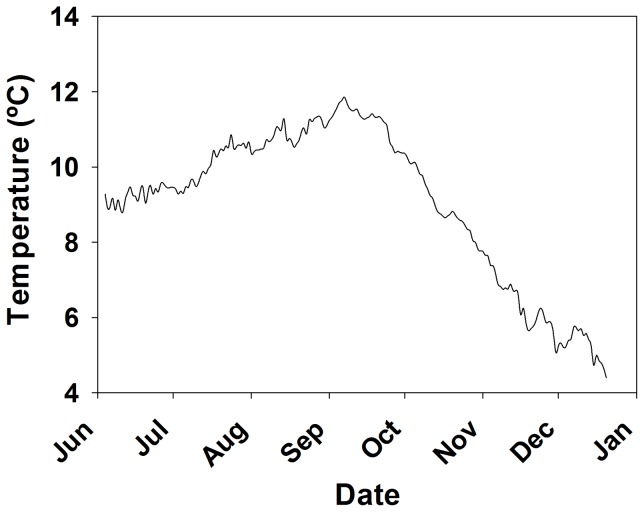
Temperatures in experimental treatments during experiment. Mean daily temperature in individual holding cells for red king crabs and Tanner crabs throughout the experiment from June to December 2010. The average daily standard deviation (not shown) was 0.1 °C.

The mean pHs±SD in the three treatments were: 8.040±0.040, 7.802±0.025, and 7.503±0.040 and they differed significantly among all treatments (ANOVA *F* = 11,435, *P*<5×10^−324^). The mean temperature for all treatments was 9.1±2.0 °C (SD) and did not vary among treatments (ANOVA *F* = 0.025, *P* = 0.975). Temperatures were 9.3 °C in June at the beginning of the experiment, rose to a high of 11.9 °C in September and fell to 4.4 °C in December at the end of the experiment (Fig. 2). As expected, alkalinity did not vary among treatments (ANOVA, *F* = 1.397, *P* = 0.254; Table 1) and DIC increased with decreasing pH (ANOVA, *F* = 68.607, *P*<0.0005; Table 1). Both aragonite and calcite were supersaturated in the Control treatment, aragonite was right at saturation and calcite was supersaturated in the pH 7.8 treatment, and both were undersaturated in the pH 7.5 treatment (Table 1).

**Table 1:** The mean and standard error (SE) of water chemistry parameters measured (DIC, and Alkalinity) and calculated (all others, see text for details) in the three treatments during the experiments.

	pH_F_	pCO_2_	HCO_3_^−^	CO_3_^−2^	DIC	Alkalinity	Ω_Aragonite_	Ω_Calcite_
Treatment		uatm	mmol/kg	mmol/kg	mmol/kg	mmol/kg		
Control	8.04	437.57	1.82	0.09	1.93	1.93	1.43	2.27
SE	0.003	8.68	0.01	0.00	0.01	0.01	0.03	0.05
pH 7.8	7.80	791.90	1.91	0.06	2.01	2.01	0.87	1.38
SE	0.002	6.82	0.01	0.00	0.01	0.01	0.01	0.02
pH 7.5	7.50	1637.71	1.97	0.03	2.08	2.08	0.44	0.71
SE	0.003	13.61	0.01	0.00	0.01	0.01	0.01	0.01

N = 23–28 per treatment for DIC and Alkalinity and N = 200 per treatment for pH_F_.

**Figure 3:**
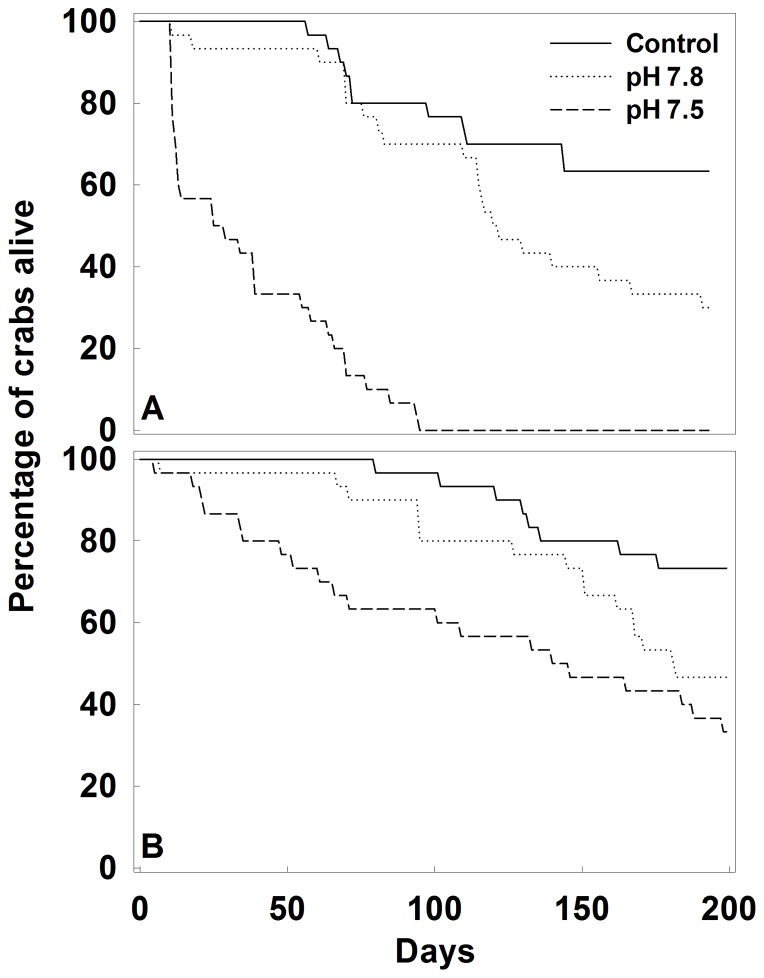
Survival of crabs in Control and Acidified water. Red king crab (A), and Tanner crabs (B) in control and treatment tanks over the duration of the experiment. Maximum likelihood estimated mortality rates±standard error were Control −0.0023±0.00007 day^−1^, pH 7.8−0.0047±0.00011 day^−1^, pH 7.5−0.025±0.00066 day^−1^ for red king crab and Control -0.0010±0.00004 day^−1^, pH 7.8-0.0023±0.00007 day^−1^, pH 7.5-0.0050±0.00011 day^−1^ for Tanner crab. See text for model details.

For both species, survival was best described by the model in which the mortality rate differed among all treatments (Table 2). Red king crab mortality rates were lowest in control water, 104% higher in pH 7.8 water, and 997% higher in pH 7.5 water (Fig. 3). Tanner crab mortality rates were also lowest in control water, 130% higher in pH 7.8 water, and 400% higher in pH 7.5 water (Fig. 3). Red king crabs were particularly vulnerable, with 100% mortality in the pH 7.5 treatment occurring after 95 days.

**Table 2:** Models of survival for red king crab and Tanner crab ranked using AIC_c_.

Model	K	AIC_c_	ΔAIC_c_	Likelihood	AIC_c_ Weights
Red king crab					
All Same	1	5591.51	3478.82	0.00	0.00
All Different	3	2112.70	0.00	1.00	1.00
Control<Acidified	2	4211.03	2098.34	0.00	0.00
Tanner crab					
All Same	1	3687.07	1454.25	0.00	0.00
All Different	3	2232.82	0.00	1.00	1.00
Control<Acidified	2	2703.17	470.34	0.00	0.00

Model indicates how the mortality rate was modeled as a function of treatment (see text for details). K indicates the number of parameters.

**Figure 4:**
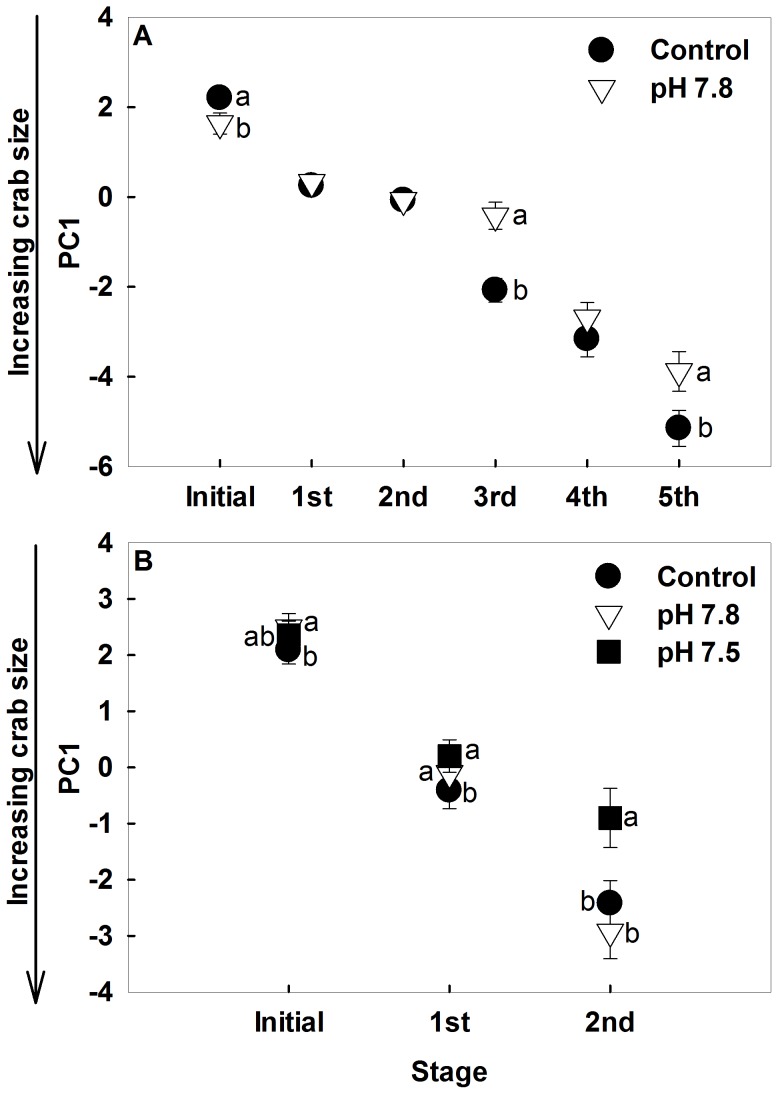
Changes in crab morphology in Control and Acidified water. Mean PC1 scores (±SE) from principle component analysis of red king crab (A) and Tanner crab (B) morphometrics for each molt for crabs held in Control, pH 7.8, and pH 7.5 water. Symbols with different letters beside them differ significantly within that molt stage. Note that crab size is inversely proportional to PC1 for both species.

Red king crabs in the Control and pH 7.8 treatments molted up to five times during the experiment providing an adequate base to assess morphometric effects. The first 2 principal components (PC) explained 94% of the variance in the red king crab morphometrics, with the first PC explaining 89% (Table 3). As so few in the pH 7.5 survived even after the first molt (only one molted a second time and it died soon after), we did not include the pH 7.5 crab in our ANOVA analysis (Table 3). The first PC was significantly different among treatments, stages, and the interaction between them and was negatively correlated with all morphometric measurements (Table 3). At the beginning of the experiment, the crabs in the pH 7.8 treatment were slightly larger than those in the Control, as evidenced by a smaller average PC1 score. However, as the experiment progressed, the Control crabs grew more per molt such that after the 3^rd^ through 5^th^ molts they were larger than the pH 7.8 crabs, though this was only significant after the 3^rd^ and 5^th^ molts (Fig. 4). The second PC was significantly different among treatments and stages, and was positively correlated with carapace length, carapace width, and orbital spine width and negatively correlated with rostrum base width and first spine length (Table 3). The mean(±standard error) PC2 scores for the Control was −0.0139±0.049 and for pH 7.8 was −0.094±0.056, indicating that control crabs had slightly different shapes than the pH 7.8 crabs.

**Table 3:** Principal component analysis of red king crab morphometrics.

PC	Eigenvalues	%Variation	Cum.%Variation
1	4.43	88.5	88.5
2	0.27	5.4	93.9
	**Eigenvectors**		
**Variable**	**PC1**	**PC2**	
Carapace width	−0.463	0.319	
Carapace length	−0.463	0.283	
Rostrum base width	−0.431	−0.449	
Orbital spine width	−0.451	0.431	
1st spine length	−0.427	−0.656	
**ANOVA**			
**Variable**	**Factor**	***F***	***P***
**PC1**	Treatment	8.507	0.004
	Molt #	191.521	<0.0005
	T*M	5.433	<0.0005
	Crab(T)	3.943	<0.0005
**PC2**	Treatment	10.842	0.001
	Molt #	6.277	<0.0005
	T*M	1.853	0.107
	Crab(T)	1.133	0.278

The first two eigenvectors representing 94% of the variance are retained. ANOVA analysis with Treatment (T), Molt number (M) as factors.

Tanner crab molted up to three times during the experiment. The first PC explained 96% of the data and so it was the only one retained. As only one of the crabs in the pH 7.5 treatment (10%), two crabs in the pH 7.8 treatment (14%), and 8 (36%) of the control crabs molted a third time, we did not include the third molt in our ANOVA analysis of PC1. The first PC was significantly different among treatments, stages, and the interaction between them and was negatively correlated to all morphometric measurements (Table 4). At the beginning of the experiment, the crabs in the pH 7.8 treatment were slightly smaller than those in the Control, while there was no difference between the Control and the pH 7.5 treatments. After the first molt, the pH 7.5 and the pH 7.8 treatment were smaller than the Control, and after the second molt, the pH 7.5 treatments were smaller than the Control and pH 7.8 (Fig. 4). This suggests that the pH 7.5 crabs were growing more slowly than the Control and pH 7.8 crabs.

**Table 4:** Principal component analysis of Tanner crab morphometrics.

PC	Eigenvalues	%Variation	Cum.%Variation
1	7.66	95.8	95.8
**Eigenvectors**			
**Variable**	**PC1**		
Carapace width	−0.359		
Carapace length	−0.36		
CL rostrum horn	−0.36		
CL eye orbit	−0.359		
Rostrum base width	−0.353		
Orbital spine width	−0.359		
Orbital spine length	−0.343		
Rostrum horn length	−0.334		
**ANOVA**			
**Variable**	**Factor**	***F***	***P***
**PC1**	Treatment	17.433	<0.0005
	Molt #	836.644	<0.0005
	T*M	3.374	0.012
	Crab(T)	19.091	<0.0005

The first eigenvector representing 96% of the variance is retained. ANOVA analysis with Treatment (T), Molt number (M) as factors.

**Figure 5:**
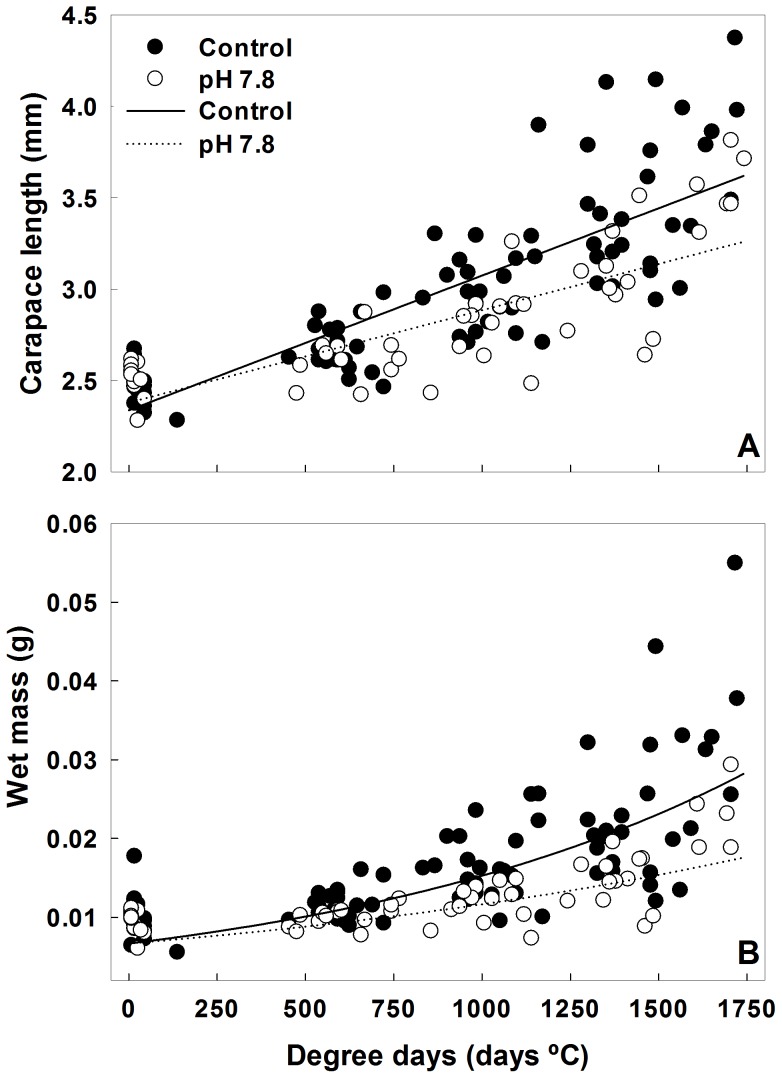
Growth of red king crab in Control and pH 7.8 water. A) Carapace length and B) Wet mass. Points represent individual measurements and lines the best fit models (see text for details. Equations for the lines are: Control-*CL = *0.00737*t+*2.34, *WM* = 0.00667*e* ^0.000829*t*^; pH 7.8-*CL* = 0.000506*t*+2.38, *WM* = 0.00667*e* ^0.000557*t*^ where *CL* is carapace length, *WM* is wet mass, and *t* is time in degree days.

Red king crabs grew faster in the control than in the pH 7.8 treatment both in terms of CL and WM (Fig. 5). There was a significant interaction between Time (in degree days) and treatment in the analysis of CL (ANCOVA, *F* = 10.653, *P* = 0.001) indicating a difference in slopes between the treatments. Each treatment was then regressed separately, with crab number included in the model as a factor (Fig. 5). The change in carapace length over time was greater in control than in pH 7.8 such that by the end of the experiment the regression equations predict 11% longer crabs in the control treatment. The best fitting model of wet mass growth had the ‘b’ parameter differing between treatments (Table 5), indicating a faster growth rate in control water (Fig. 5). By the end of the experiment the model predicted that crabs in control water had 61% higher masses than crabs in pH 7.8 water. The average (±SD) intermolt period for red king crab was 450±119 degree days and ranged from 172 to 812 degree days.

**Table 5:** Models of red king crab growth ranked using AIC_c_.

Model	K	AIC_c_	ΔAIC_c_	Likelihood	AIC_c_ Weights
*a*, *b*	38	−1210.96	12.29	0.00	0.00
*a*(T), *b*	38	−1205.12	18.13	0.00	0.00
*a*, *b*(T)	39	−1223.25	0.00	1.00	1.00
*a*(T), *b*(T)	39	−1211.12	12.13	0.00	0.00

Model indicates how the two parameters, *a* and *b*, were modeled. In all models *a* was allowed to vary linearly with crab number (nested within treatment). ‘T’ indicates that the parameter was allowed to vary with pH treatment. See text for model details.

**Figure 6:**
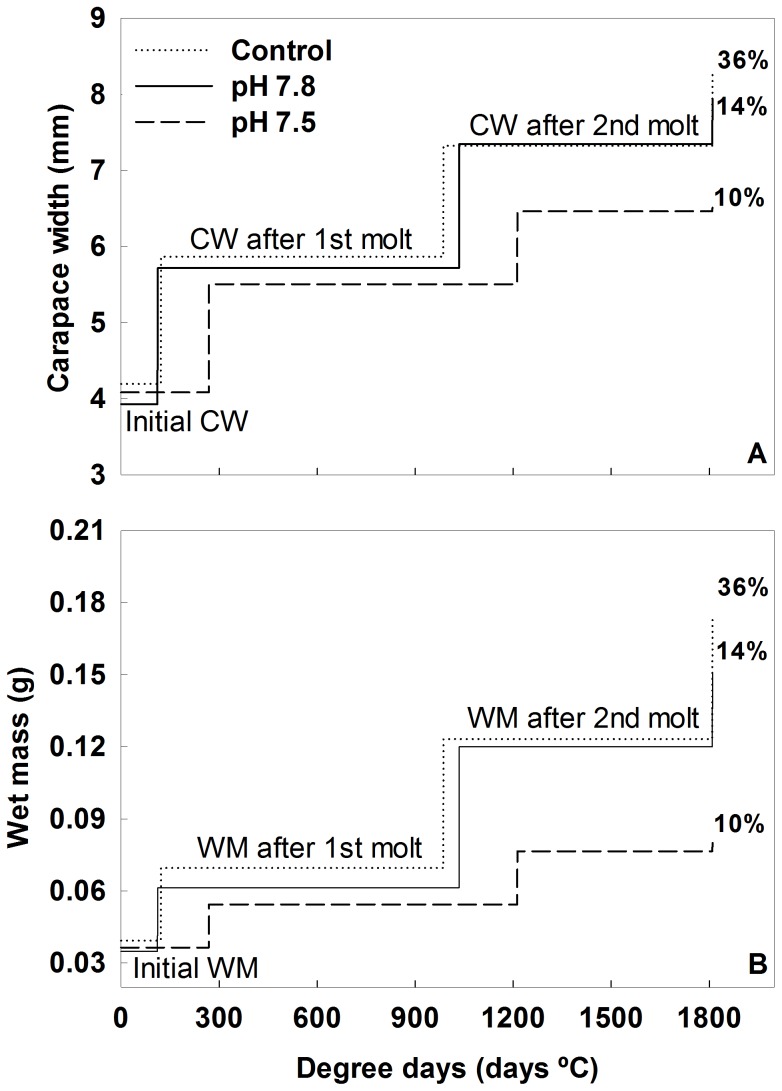
Growth of Tanner crab in Control and Acidified water. A) carapace width and B) wet mass. Lines represent the average CW or wet mass after each molt and the distance between the vertical jumps indicates the average intermolt duration. The height of the final line represents the average CW or wet mass at the end of the experiment and the number indicates the percentage of crabs molting a third time in each of the treatments.

Tanner crabs grew faster in Control and pH 7.8 water than in pH 7.5 water. At the beginning of the experiment, there was no difference in the CW (ANOVA, *F* = 0.735, *P* = 0.482) or WM (ANOVA, *F* = 0.576, *P* = 0.564). This lack of a difference persisted after the first molt (CW, KW test statistic = 1244, *P* = 0.537; WM, KW test statistic = 2.832, *P* = 0.243) and second molt (CW, KW test statistic = 2.182, *P* = 0.336; WM, KW test statistic = 4.206, *P* = 0.122). The average (±SD) intermolt period for Tanner crab was 873±198 degree days and ranged from 589 to 1481 degree days. Intermolt period did not differ between the start and the first molt (KW test statistic = 1.73, *P* = 0.421), and first and second molts (KW test statistic = 3.361, *P* = 0.186). However, there was a consistent trend for longer intermolt periods and smaller growth increments in the pH 7.5 water (Fig. 6), such that by the end of the experiment there was a significant effect of treatment on both CW (KW test statistic = 8.185, *P* = 0.017) and WM (KW test statistic = 8.302, *P* = 0.015; Fig. 6); crabs in control water had 5% larger CWs and 16% larger WMs than crabs in pH 7.8 water, and had 28% larger CWs and 118% larger WMs than crabs in pH 7.5 water.

**Figure 7:**
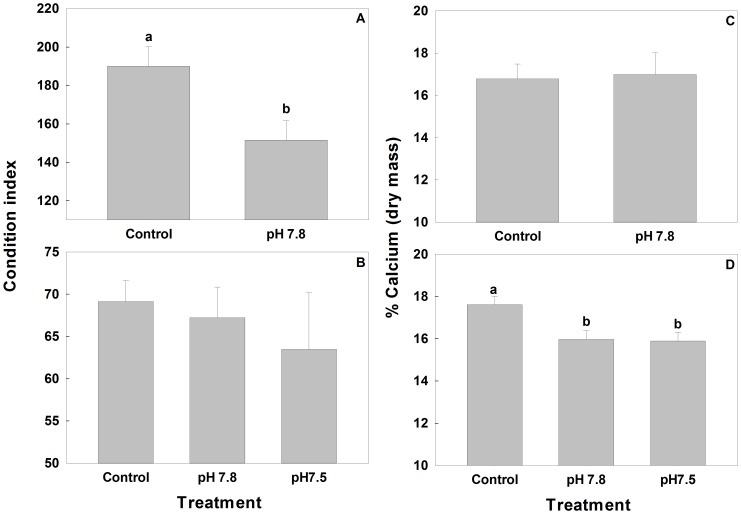
Condition index and calcification in crabs held in Control and Acidified water. Condition index of red king crabs (A) and Tanner crabs (B) and percent calcium (dry mass) in red king crabs (C) and Tanner crabs (D) at the end of the experiment. Bars are mean+SE. Bars with different letters above them differ significantly.

The condition index of RKC at the end of the experiment was 25% higher in Control treatment than in pH 7.8 (ANOVA, *F* = 5.388, *P* = 0.028; Fig. 7a). Percent calcium (dry mass) in RKC did not differ between the treatments (*F* = 0.028, *P* = 0.868; Fig. 7c). Percent magnesium (dry mass) in RKC averaged (±SD) 0.83±0.03% and also did not differ between the treatments (*F* = 1.474, *P* = 0.236). Tanner crab showed no difference among treatments in condition index (*F* = 0.509, *P* = 0.605; Fig. 7b) or percent magnesium which averaged (±SD) 1.2±0.1% dry mass (*F* = 2.572, *P* = 0.088). Percent calcium was higher in Control crabs than in pH 7.8 or pH 7.5 crabs by 10% and 11% respectively (*F* = 5.868, *P* = 0.006, Fig. 7d).

## Discussion

Ocean acidification substantially reduced the growth, condition, and survival of two phylogenetically diverse crab species in this long-term experiment. Even a modest decline of ∼0.2 pH units, a decline expected within the next century, had significant effects on both species. Given the mortality and growth rates, ocean acidification levels predicted within a century will likely cause a significant decline in the populations of both species in the absence of phenotypic or evolutionary adaptation.

The most serious negative effect of ocean acidification in this study was to increase the mortality rate in both species tested. Red king crabs were particularly vulnerable, with all crabs dying within 95 days at pH 7.5. This mirrors the increased mortality rate of red king crab larvae in low pH water [46]. Similar increases in mortality occur for some crustaceans [27], [54], [55] but not others [17], [56], [57]. The increase in mortality was evident almost immediately for both species at pH 7.5, but there was not a notable difference in mortality between the Control and pH 7.8 treatments for 50–70 days. Such a delay in the response also occurs in the shrimp *Palaemon pacificus* [27] and may be due to the ability of crustaceans to regulate their acid-base equilibrium better than other species in the short term, but at an energetic cost that eventually results in mortality when energy reserves are depleted. This result, combined with our other results on growth and calcification, suggests that short-term exposure to moderate decreases in pH would have no long-term effect on these crab species. A similar pattern occurs with estuarine animals exposed to short-term acidification [58]. Future experiments exposing animals to pHs that vary seasonally as they do in the field [59] should be performed to examine how periodicity in pH affects survival.

The morphometry of both crab species was not affected by pH even after several molts. Although red king crabs in pH 7.8 did have a lower PC2 score than control crabs, indicating slightly different shape, the difference was so small, with the PC2 explaining only about 5% of the variance, that the absolute difference is unlikely to be biologically meaningful. Other species do exhibit such morphological changes; larval pteropods, *Cavolinia inflexa*, exhibit malformed shells at a pH of 7.82 [14] and larval oysters, *Crassostrea gigas*, fail to develop normally at a pH of 7.4 [60]; however, rapid development at the larval stage may make them more vulnerable to stressors. Red king crab embryos and larvae do exhibit slight differences in morphology when exposed to low pH, but the major difference is that the embryos and larvae held at low pH are slightly larger than those held in control water [46].

Both species grew more slowly in low pH waters. Again, red king crabs were more sensitive, showing a significant difference between the pH 7.8 and control treatments, whereas for Tanner crabs the effect was not significant until the pH was reduced to 7.5. A similar decrease in growth with decreasing pH occurs in some crustaceans [17], [27], [61], but not others [57], [62]. More subtly, the condition index of red king crabs, though not Tanner crabs, was decreased under acidified conditions, and calcium content was decreased in Tanner crabs but not in red king crabs. This suggests that red king crab and Tanner crab differ in their physiological responses. Whiteley [63] suggests that crustaceans that are good ionoregulators or osmoregulators may experience a significant energetic cost when exposed to acidified waters. For example, the velvet swimming crab *Necora puber* is able to buffer its hemolymph in acidified seawater and not suffer any shell dissolution; however, at the same time it undergoes metabolic depression which likely reduces growth [64] We hypothesize that Tanner crab do not substantially increase their expenditure of energy in ionoregulation or osmoregulation or calcification in response to decreased pH, as evidenced by lower calcium content combined with a comparatively modest reduction in growth and no significant decrease in condition index. Red king crabs, similar to other crustacean species [19], seem to expend more energy in osmoregulation and calcification under acidified conditions, as evidenced by either an increased calcium content in larvae and adults [46], or a maintenance of calcium content in juveniles. As in other species [6], this comes at the cost of decreased growth, and condition, as the animals have less energy to expend on growth. The same mechanism is implicated to explain decreased growth and survival in barnacle, *Amphibalanus improvisus*, larvae [65] and in marine shrimp *Palaemon pacidficus* [27] as a result of increased CO_2_.

The calcification of the crabs in our study has implications for our understanding of the effects of ocean acidification on calcification in crustaceans. As reviewed by Whiteley [63], the short to medium-term effect of reduced pH is typically either an increase in calcification or no net effect (e.g. [19], [66], [67]). In this long-term experiment, Tanner crab decreased calcification in low pH water, something observed in few other crustacean species; Walther [13] observed a decrease in calcification in *Hyas araneus* larvae and Kurihara et al. [27] inferred lower calcification rates in *Palaemon pacificus* from a decrease in antenna length. Red king crab may differ in their short-term and long-term responses. In the short term, both larvae and adults increase their calcification [46], whereas in this study there was no increase in calcification in the long-term, although this may be due to life-history differences.

Though the increase in mortality at the lowest pH levels would obviously have an effect on the populations of both species, sublethal effects at more moderate, and therefore more imminent, reductions in pH will likely affect population and community level processes. Smaller crab are more vulnerable to predators [68] and do not satiate predators as quickly [29], and thus a decrease in growth rate is likely to reduce the survival of a cohort to maturity. Similarly, lower calcium content may make crabs easier prey for crushing predators. In addition, lower growth rates will either increase the time it takes for crabs to reach maturity or decrease the size at maturity, smaller females are less fecund for both species [38], [69], and individuals with a lower condition index may have a lower fecundity [53], and so slower growth and a lower condition index are likely to reduce the lifetime egg production of females. Lower survival and lifetime fecundity and changes in predator-prey dynamics [70], [71] are likely to negatively affect the populations of both species and therefore the benthic community. At the community level [72] the effect of ocean acidification is likely to be similar to that of other, better studied marine stressors such as hypoxia; species-specific tolerances [73] and behavioral changes [74], [75] alter the outcomes of interspecific interactions [76], [77] leading to predictable changes in community structure [78]–[80].

Levels of ocean acidification expected within a century are likely to have a profound effect on the two economically important crab species in this study. Increases in mortality and decreases in growth and condition suggest that the populations of both species will suffer, with red king crab being affected first. However, it is currently unknown how much phenotypic plasticity [81] and evolutionary adaptation [82], which occur in other species, may enable these crabs to survive, although the length of this study suggests that phenotypic plasticity may not have an appreciable effect for these species. The change in pH over time is predicted to be gradual, so populations will have some time to respond. Both species have closely related species, such as the scarlet king crab, *Lithodes cousi*, and the grooved Tanner crab, *Chionoecetes tanneri*, that live in and are adapted to deep, low pH waters [23], [83], [84], suggesting that such adaptation is possible. However, as pH levels drop in surface waters, these deep-sea crabs may begin to compete successfully for shallower-water habitat. Finally, this research highlights the need for long-term exposure experiments to accurately predict the effects of ocean acidification. In our experiment increases in mortality and decreases in growth were not apparent during the first 3–4 months of exposure. Future research should focus on understanding the effects of ocean acidification at all life history stages for both species and on effects on intraspecific interactions under acidified conditions.

## References

[1] Raven J, Caldeira K, Elderfield H, Hoegh-Guldberg O, Liss P, et al.. (2006) Ocean acidification due to increasing atmospheric carbon dioxide. The Royal Society Policy Document 12 5.

[2] Feely RA, Sabine CL, Lee K, Berelson W, Kleypas J, et al (2004) Impact of anthropogenic CO_2_ on the CaCO_3_ system in the oceans. Science 305: 362–366. 15256664 10.1126/science.1097329

[3] Caldeira K, Wickett ME (2003) Anthropogenic carbon and ocean pH. Nature 425: 365–365. 14508477 10.1038/425365a

[4] Doney SC, Fabry VJ, Feely RA, Kleypas JA (2009) Ocean acidification: The other CO_2_ problem. Annual Review of Marine Science 1: 169–192. 10.1146/annurev.marine.010908.163834 21141034

[5] Kroeker KJ, Kordas RL, Crim RN, Singh GG (2010) Meta-analysis reveals negative yet variable effects of ocean acidification on marine organisms. Ecology Letters 13: 1419–1434. 20958904 10.1111/j.1461-0248.2010.01518.x

[6] Wood HL, Spicer JI, Widdicombe S (2008) Ocean acidification may increase calcification rates, but at a cost. Proceedings of the Royal Society B-Biological Sciences 275: 1767–1773. 10.1098/rspb.2008.0343 PMC2587798 18460426

[7] Martin S, Gattuso JP (2009) Response of Mediterranean coralline algae to ocean acidification and elevated temperature. Global Change Biology 15: 2089–2100.

[8] Comeau S, Jeffree R, Teyssie JL, Gattuso JP (2010) Response of the arctic pteropod *Limacina helicina* to projected future environmental conditions. PLoS ONE 5: e11362. 20613868 10.1371/journal.pone.0011362 PMC2894046

[9] Gao KS, Zheng YQ (2010) Combined effects of ocean acidification and solar UV radiation on photosynthesis, growth, pigmentation and calcification of the coralline alga *Corallina sessilis* (Rhodophyta). Global Change Biology 16: 2388–2398.

[10] Parker LM, Ross PM, O'Connor WA (2009) The effect of ocean acidification and temperature on the fertilization and embryonic development of the Sydney rock oyster *Saccostrea glomerata* (Gould 1850). Global Change Biology 15: 2123–2136.

[11] Watson S, Southgate P, Tyler P, Peck L (2009) Early larval development of the Sydney rock oyster *Saccostrea glomerata* under near-future predictions of CO_2_-driven ocean acidification. Journal of Shellfish Research 28: 431–437.

[12] Talmage SC, Gobler CJ (2009) The effects of elevated carbon dioxide concentrations on the metamorphosis, size, and survival of larval hard clams (*Mercenaria mercenaria*), bay scallops (*Argopecten irradians*), and Eastern oysters (*Crassostrea virginica*). Limnology and Oceanography 54: 2072–2080.

[13] Walther K, Anger K, Portner HO (2010) Effects of ocean acidification and warming on the larval development of the spider crab *Hyas araneus* from different latitudes (54 degrees vs. 79 degrees N). Marine Ecology Progress Series 417: 159–170.

[14] Comeau S, Gorsky G, Alliouane S, Gattuso JP (2010) Larvae of the pteropod *Cavolinia inflexa* exposed to aragonite undersaturation are viable but shell-less. Marine Biology 157: 2341–2345.

[15] Parker LM, Ross PM, O'Connor WA (2010) Comparing the effect of elevated pCO_2_ and temperature on the fertilization and early development of two species of oysters. Marine Biology 157: 2435–2452.

[16] Ellis RP, Bersey J, Rundle SD, Hall-Spencer JM, Spicer JI (2009) Subtle but significant effects of CO_2_ acidified seawater on embryos of the intertidal snail, *Littorina obtusata* . Aquatic Biology 5: 41–48.

[17] Arnold KE, Findlay HS, Spicer JI, Daniels CL, Boothroyd D (2009) Effect of CO_2_-related acidification on aspects of the larval development of the European lobster, *Homarus gammarus* (L.). Biogeosciences 6: 1747–1754.

[18] Havenhand JN, Schlegel P (2009) Near-future levels of ocean acidification do not affect sperm motility and fertilization kinetics in the oyster *Crassostrea gigas* . Biogeosciences 6: 3009–3015.

[19] Ries JB, Cohen AL, McCorkle DC (2009) Marine calcifiers exhibit mixed responses to CO_2_−induced ocean acidification Geology. 37: 1131–1134.

[20] Byrne M, Soars N, Selvakumaraswamy P, Dworjanyn SA, Davis AR (2010) Sea urchin fertilization in a warm, acidified and high pCO_2_ ocean across a range of sperm densities. Marine Environmental Research 69: 234–239. 19913293 10.1016/j.marenvres.2009.10.014

[21] Dupont S, Lundve B, Thorndyke M (2010) Near future ocean acidification increases growth rate of the lecithotrophic larvae and juveniles of the sea star *Crossaster papposus* . Journal of Experimental Zoology Part B-Molecular and Developmental Evolution 314B: 382–389. 10.1002/jez.b.21342 20309996

[22] Dupont S, Moya A, Bailly X (2012) Stable photosymbiotic relationship under CO_2_-induced acidification in the acoel worm *Symsagittifera roscoffensis* . PLoS ONE 7: e29568. 22253736 10.1371/journal.pone.0029568 PMC3253794

[23] Pane EF, Barry JP (2007) Extracellular acid-base regulation during short-term hypercapnia is effective in a shallow-water crab, but ineffective in a deep-sea crab. Marine Ecology Progress Series 334: 1–9.

[24] Beesley A, Lowe DM, Pascoe CK, Widdicombe S (2008) Effects of CO_2_-induced seawater acidification on the health of *Mytilus edulis* . Climate Research 37: 215–225.

[25] Bibby R, Cleall-Harding P, Rundle S, Widdicombe S, Spicer J (2007) Ocean acidification disrupts induced defences in the intertidal gastropod *Littorina littorea* . Biology Letters 3: 699–701. 17939976 10.1098/rsbl.2007.0457 PMC2391239

[26] Comeau S, Gorsky G, Jeffree R, Teyssie JL, Gattuso JP (2009) Impact of ocean acidification on a key Arctic pelagic mollusc (*Limacina helicina*). Biogeosciences 6: 1877–1882.

[27] Kurihara H, Matsui M, Furukawa H, Hayashi M, Ishimatsu A (2008) Long-term effects of predicted future seawater CO_2_ conditions on the survival and growth of the marine shrimp *Palaemon pacificus* . Journal of Experimental Marine Biology and Ecology 367: 41–46.

[28] Donaldson W, Byersdorfer S (2005) Biological field techniques for lithodid crabs. University of Alaska Fairbanks.

[29] Long WC, Popp J, Swiney KM, Van Sant SB (2012) Cannibalism in red king crab, *Paralithodes camtschaticus* (Tilesius, 1815): Effects of habitat type and predator density on predator functional response. Journal of Experimental Marine Biology and Ecology 422–423: 101–106.

[30] Stoner AW (2009) Habitat-mediated survival of newly settled red king crab in the presence of a predatory fish: Role of habitat complexity and heterogeneity. Journal of Experimental Marine Biology and Ecology 382: 54–60.

[31] Jadamec L, Donaldson W, Cullenberg P (1999) Biological field techniques for *Chionoecetes* crabs. University of Alaska Sea Grant. AK-SG-99-02, Fairbanks.

[32] Stevens BG, Swiney KM (2007) Hatch timing, incubation period, and reproductive cycle for captive primiparous and multiparous red king crab, *Paralithodes camtschaticus* . Journal of Crustacean Biology 27: 37–48.

[33] Swiney KM (2008) Egg extrusion, embryo development, timing and duration of eclosion, and incubation period of primiparous and multiparous tanner crabs (*Chionoecetes bairdi*). Journal of Crustacean Biology 28: 334–341.

[34] Shirley SM, Shirley TC (1989) Interannual veriability in density, timing and survival of Alaskan red king crab *Paralithodes camtschatica* larvae. Marine Ecology Progress Series 54: 51–59.

[35] Donaldson WE, Cooney RT, Hilsinger JR (1981) Growth, age and size at maturity of Tanner crab, *Chionoecetes bairdi* M. J. Rathbun, in the northern Gulf of Alaska (Decapoda, Brachyura). Crustaceana 40: 286–302.

[36] Stevens BG (1990) Temperature-dependent growth of juvenile red king crab (*Paralithodes camtschatica*) and its effects on size-at-age and subsequent recruitment in the Eastern Bering Sea. Canadian Journal of Fisheries and Aquatic Sciences 47: 1307–1317.

[37] Donaldson W, Byersdorfer S, Pengilly D, Blau S (1992) Growth of red king crab, Paralithodes camtschaticus (Tilesius, 1815), in artificial habitat collectors at Kodiak, Alaska. Journal of Shellfish Research 11: 85–89.

[38] Somerton DA, Meyers WS (1983) Fecundity differences between primiparous and multiparous female Alaskan Tanner crab (Chionoecetes bairdi). Journal of Crustacean Biology 3: 183–186.

[39] Tamone SL, Taggart SJ, Andrews AG, Mondragon J, Nielsen JK (2007) The relationship between circulating ecdysteroids and chela allometry in male tanner crabs: Evidence for a terminal molt in the genus Chionoecetes. Journal of Crustacean Biology 27: 635–642.

[40] NPFMC (2011) Stock assessment and fishery evaluation report for the king and Tanner crab fisheries of the Bering Sea and Aleutian Islands regions. Anchorage, AK: North Pacific Fishery Management Council. 677 p.

[41] Stevens BG, Swiney KM (2007) Growth of female red king crabs *Paralithodes camtshaticus* from Kodiak, Alaska, during pubertal, primiparous, and multiparous molts. Alaska Fishery Research Bulletin 12: 270–277.

[42] Hoopes D, Karinen J (1972) Longevity and growth of tagged king crabs in the Eastern Bering Sea. Fish Bull Nat Oceanic Atmos Admin 70: 225–226.

[43] Skinner DM (1985) Molting and regeneration. In: Bliss DE, Mantel LH, editors. Integument, Pigments, and Hormonal Processes. Orlando: Academic Press, Inc. pp. 44–146.

[44] Stevens BG (2009) Hardening of red king crab *Paralithodes camtschaticus* (Tilesius, 1815) shells after molting. Journal of Crustacean Biology 29: 157–160.

[45] Fabry VJ, McClintock JB, Mathis JT, Grebmeier JM (2009) Ocean acidification at high latitudes: The bellweather. Oceanography 22: 160–171.

[46] Long WC, Swiney KM, Foy RJ (In press) Effects of ocean acidification on the embryos and larvae of red king crab, *Paralithodes camtschaticus*. Marine Pollution Bulletin 10.1016/j.marpolbul.2013.01.011. 10.1016/j.marpolbul.2013.01.011 23434384

[47] McGraw CM, Cornwall CE, Reid MR, Currie KI, Hepburn CD, et al (2010) An automated pH-controlled culture system for laboratory-based ocean acidification experiments. Limnology and Oceanography-Methods 8: 686–694.

[48] Swiney KM, Long WC, Persselin SL The effects of holding space on juvenile red king crab (*Paralithodes camtschaticus)* growth and survival. Aquaculture Research 10.1111/j.1365–2109.2012.03105.x. In press.

[49] Millero FJ (1986) The pH of estuarine waters. Limnology and Oceanography: 839–847.

[50] Dickson AG, Sabine CL, Christian JR (2007) Guide to best practices for ocean CO_2_ measurements. PICES special publication 3. 191 p.

[51] Lavigne H, Gattuse J (2012) seacarb: seawater carbonate chemistry with R. Available: http://CRAN.R-project.org/package=seacarb. R package version 2.4.6 ed.

[52] Burnham KP, Anderson DR (2002) Model selection and multimodel inference: A practical information-theoretic approach. New York: Springer Science+Business Media. 488 p.

[53] Long WC, Bromage E, Seitz RD, Kaattari S (2008) Quantifying fecundity in *Macoma balthica* using an enzyme-linked immunosorbent assay (ELISA). Aquatic Biology 3: 187–193.

[54] Donohue PJC, Calosi P, Bates AH, Laverock B, Rastrick S, et al (2012) Impact of exposure to elevated pCO_2_ on the physiology and behaviour of an important ecosystem engineer, the burrowing shrimp *Upogebia deltaura* . Aquatic Biology 15: 73–86.

[55] Walther K, Sartoris F, Bock C, Pörtner H (2009) Impact of anthropogenic ocean acidification on thermal tolerance of the spider crab *Hyas araneus* . Biogeosciences 6: 2207–2215.

[56] Kurihara H, Ishimatsu A (2008) Effects of high CO_2_ seawater on the copepod (*Acartia tsuensis*) through all life stages and subsequent generations. Marine Pollution Bulletin 56: 1086–1090. 18455195 10.1016/j.marpolbul.2008.03.023

[57] Findlay HS, Kendall MA, Spicer JI, Widdicombe S (2010) Post-larval development of two intertidal barnacles at elevated CO_2_ and temperature. Marine Biology 157: 725–735.

[58] Amaral V, Cabral HN, Bishop MJ (2011) Resistance among wild invertebrate populations to recurrent estuarine acidification. Estuarine Coastal and Shelf Science 93: 460–467.

[59] Mathis JT, Cross JN, Bates NR (2011) Coupling primary production and terrestrial runoff to ocean acidification and carbonate mineral suppression in the eastern Bering Sea. Journal of Geophysical Research-Oceans 116: 24.

[60] Kurihara H, Kato S, Ishimatsu A (2007) Effects of increased seawater pCO_2_ on early development of the oyster *Crassostrea gigas* . Aquatic Biology 1: 91–98.

[61] Fitzer SC, Caldwell GS, Close AJ, Clare AS, Upstill-Goddard RC, et al (2012) Ocean acidification induces multi-generational decline in copepod naupliar production with possible conflict for reproductive resource allocation. Journal of Experimental Marine Biology and Ecology 418: 30–36.

[62] Hauton C, Tyrrell T, Williams J (2009) The subtle effects of sea water acidification on the amphipod *Gammarus locusta* . Biogeosciences 6: 1479–1489.

[63] Whiteley NM (2011) Physiological and ecological responses of crustaceans to ocean acidification. Marine Ecology Progress Series 430: 257–271.

[64] Small D, Calosi P, White D, Spicer JI, Widdicombe S (2010) Impact of medium-term exposure to CO_2_ enriched seawater on the physiological functions of the velvet swimming crab *Necora puber* . Aquatic Biology 10: 11–21.

[65] Pansch C, Nasrolahi A, Appelhans YS, Wahl M (2012) Impacts of ocean warming and acidification on the larval development of the barnacle *Amphibalanus improvisus* . Journal of Experimental Marine Biology and Ecology 420: 48–55.

[66] McDonald MR, McClintock JB, Amsler CD, Rittschof D, Angus RA, et al (2009) Effects of ocean acidification over the life history of the barnacle *Amphibalanus amphitrite* . Marine Ecology Progress Series 385: 179–187.

[67] Findlay HS, Kendall MA, Spicer JI, Widdicombe S (2009) Future high CO_2_ in the intertidal may compromise adult barnacle *Semibalanus balanoides* survival and embryonic development rate. Marine Ecology Progress Series 389: 193–202.

[68] Pirtle JL, Eckert GL, Stoner AW (2012) Habitat structure influences the survival and predator-prey interactions of early juvenile red king crab *Paralithodes camtschaticus* . Marine Ecology Progress Series 465: 169–184.

[69] Swiney KM, Long WC, Eckert GL, Kruse GH (2012) Red king crab, *Paralithodes camtschaticus,* size-fecundity relationship, and inter-annual and seasonal variability in fecundity. Journal of Shellfish Research 31: 925–933.

[70] Amaral V, Cabral HN, Bishop MJ (2012) Effects of estuarine acidification on predator-prey interactions. Marine Ecology-Progress Series 445: 117–127.

[71] Appelhans YS, Thomsen J, Pansch C, Melzner F, Wahl M (2012) Sour times: seawater acidification effects on growth, feeding behaviour and acid-base status of *Asterias rubens* and *Carcinus maenas* . Marine Ecology Progress Series 459: 85–98.

[72] Hale R, Calosi P, McNeill L, Mieszkowska N, Widdicombe S (2011) Predicted levels of future ocean acidification and temperature rise could alter community structure and biodiversity in marine benthic communities. Oikos 120: 661–674.

[73] Modig H, Ólafsson E (1998) Responses of Baltic benthic invertebrates to hypoxic events. Journal of Experimental Marine Biology and Ecology 229: 133–148.

[74] Long WC, Brylawski BJ, Seitz RD (2008) Behavioral effects of low dissolved oxygen on the bivalve *Macoma balthica* . Journal of Experimental Marine Biology and Ecology 359: 34–39.

[75] Bell G, Eggleston D (2005) Species-specific avoidance responses by blue crabs and fish to chronic and episodic hypoxia. Marine Biology 146: 761–770.

[76] Long WC, Seitz RD (2008) Trophic interactions under stress: hypoxia enhances foraging in an estuarine food web. Marine Ecology Progress Series 362: 59–68.

[77] Altieri AH (2008) Dead zones enhance key fisheries species by providing predation refuge. Ecology 89: 2808–2818. 18959318 10.1890/07-0994.1

[78] Long WC, Seitz RD (2009) Hypoxia in Chesapeake Bay tributaries: Worsening effects on macrobenthic community structure in the York River. Estuaries and Coasts 32: 287–297.

[79] Seitz RD, Dauer DM, Llanso RJ, Long WC (2009) Broad-scale effects of hypoxia on benthic community structure in Chesapeake Bay, USA. Journal of Experimental Marine Biology and Ecology 381: S4–S12.

[80] Lenihan HS, Peterson CH, Byers JE, Grabowski JH, Thayer GW, et al (2001) Cascading of habitat degradation: oyster reefs invaded by refugee fishes escaping stress. Ecological Applications 11: 764–782.

[81] Dupont S, Dorey N, Stumpp M, Melzner F, Thorndyke M (2012) Long-term and trans-life-cycle effects of exposure to ocean acidification in the green sea urchin *Strongylocentrotus droebachiensis*. Marine Biology. Doi:10.1007/s00227-012-1921-x: 1–9

[82] Parker LM, Ross PM, O'Connor WA (2011) Populations of the Sydney rock oyster, *Saccostrea glomerata*, vary in response to ocean acidification. Marine Biology 158: 689–697.

[83] Somerton D (1981) Contribution to the life history of the deep-sea king crab, *Lithodes couesi*, in the Gulf of Alaska. Fishery Bulletin 79: 259–269.

[84] Byrne RH, Mecking S, Feely RA, Liu X (2010) Direct observations of basin-wide acidification of the North Pacific Ocean. Geophysical Research Letters 37: L02601.

